# Thinking Transtheoretically About Alliance and Rupture: Implications for Practice and Training

**DOI:** 10.32872/cpe.12439

**Published:** 2024-04-26

**Authors:** Sigal Zilcha-Mano, J. Christopher Muran

**Affiliations:** 1 Department of Psychology, University of Haifa, Haifa, Israel; 2 Gordon F. Derner School of Psychology, Adelphi University, New York, NY, USA; Department of Psychology, University of Trier, Trier, Germany

**Keywords:** rupture and repair, alliance, mechanism of change, trait-like, state-like

## Abstract

Repairing alliance ruptures has the potential to serve as a powerful mechanism of change in psychotherapy. In this article, a transtheoretical individual-specific framework for repairing alliance ruptures is proposed. According to the proposed framework, at the intake session, the therapist evaluates the trait-like tendencies of individual patients to face ruptures in interpersonal relationships. We propose a typology based on which patients are assigned to one of the following therapeutic strategies: (a) a treatment where alliance rupture and repair is the main mechanism of change (Type A), (b) an added module that augments another treatment, focusing on rupture and repair (Type B), or (c) treatment where no rupture resolution work is carried out (Type C). The proposed framework is based on cumulative clinical knowledge, and its validity and utility need to be assessed in future research.

## Highlights

- Repairing alliance ruptures is considered an important mechanism of change in the psychotherapeutic process.
- A transtheoretical and individual-specific framework for repairing alliance ruptures is proposed.
- Based on an individual’s trait-like tendencies to face ruptures in interpersonal settings, the therapist may choose among three different therapeutic approaches.

The alliance used to be an “analytic construct,” developed to address the real and practical aspects of the patient-therapist interaction (Greenson, 1967), but it eventually took on transtheoretical relevance. This was in large part due to Bordin’s (1979, 1994) reformulation, which defined it as composed of purposeful collaboration (agreement on tasks and goals) and affective bond (mutual trust and respect) between patient and therapist. It is now widely considered an integral “common factor” (Wampold & Imel, 2015), a “principle of change” (Castonguay et al., 2019), and a “quintessential integrative variable” (Wolfe & Goldfried, 1988). In contrast to previous conceptualizations, Bordin placed greater emphasis on mutuality rather than adherence to the therapist’s agenda or organizing capacity, and pointed out the inextricable tie between therapist technique and the therapeutic relationship. Bordin’s reformulation provided the basis for an “intersubjective elaboration” by Safran and Muran (2000, 2006), which recognized the negotiation between patient and therapist regarding motivational needs for agency and communion. Bordin’s definition describes the “explicit collaboration” between patient and therapist; Safran and Muran’s describes the “implicit negotiation” (see Muran, 2022).

Decades of empirical research support the importance of the patient-therapist alliance for successful treatment, regardless of theoretical orientation. Four of the most commonly used measures for assessing the alliance are (Flückiger et al., 2018): the California Psychotherapy Alliance Scale (CALPAS; Gaston & Marmar, 1991), the Helping Alliance Questionnaire (HAQ; Luborsky et al., 1996), the Vanderbilt Psychotherapy Process Scale (VPPS; Suh et al., 1989), and the Working Alliance Inventory (WAI; Horvath & Greenberg, 1986). The alliance can be assessed based on patient or therapist reports, or may be rated by an external observer watching videotaped sessions or reading session transcripts.

The various alliance measures developed over the years have been significantly informed by Bordin’s conceptualization. A recent meta-analysis suggests that a stronger alliance is significantly associated with better treatment outcome, and a weakened alliance can lead to premature termination of treatment (Flückiger et al., 2018). Indeed, the significant alliance-outcome correlation is among the most replicated findings in psychotherapy research, perhaps in psychology science in general. There is also a growing body of evidence suggesting a potential causal relation of the alliance to outcome, supporting the consideration of the alliance as a “change mechanism” (e.g., Barber et al., 2000; Zilcha-Mano, 2017; Zilcha-Mano et al., 2019; Zilcha-Mano & Fisher, 2022).

To translate this empirical knowledge into clinical practice methods of improving alliance to achieve better treatment outcome, it is critical to address the question of whether the alliance is a facilitative condition for change or a change mechanism in itself. Correlational research efforts support the former but other work suggests the latter, including mediational, within-individual effects, and task analyses. Change can be understood as involving movement on multiple levels or including multiple processes, and as noted, alliance has been defined as inherently integrated in all change processes of which collaboration and trust are a part. As a change process, the alliance can be understood to involve both skill development and a new relational or corrective experience, that is, how to negotiate one’s motivational needs with another and how to be intimate with another (expressive and understood).

One of the most studied and supported perspectives of the alliance as a change mechanism concerns rupture repair. Ruptures have been defined as (a) disagreements about tasks or goals or deterioration in trust or respect (Bordin’s formulation) and (b) breakdowns in the negotiation of the patient’s and therapist’s implicit needs for agency or communion according to Safran and Muran’s elaboration (see Safran & Muran, 2000, 2006). More specifically, ruptures have been defined based on patient or therapist markers of withdrawal or confrontation: withdrawal markers are “movements away” from self or the other that can be understood as pursuits of communion at the expense of agency (e.g., shutting down, avoiding, masking experience); confrontation markers are “movements against” the other that can be understood as pursuits of agency at the expense of communion (e.g., complaining about the other, defending the self, controlling the other; Eubanks, Muran, & Samstag, 2023; Muran & Eubanks, 2020a). At the heart of the rupture is the negotiation of individual differences and relationship ambiguities, which result in objectifications to control and feel agentic, but also reflect a struggle to recognize respective subjectivities, define oneself, and feel connected (Muran, 2007; Muran & Eubanks, 2020b).

A recent meta-analysis on rupture repair (Eubanks et al., 2019) that included self-report and observer-based methods demonstrated a similar significant relation between rupture repair episodes and treatment outcome (as ratings of the alliance quality). Several task analytic studies provided empirical support for stage-process models that define rupture repair as a change event: one example defines an exploratory model in which ruptures are acknowledged and explored and implicit needs clarified as expressions of resolution (Safran & Muran, 1996); another defines a renegotiation of tasks or goals, which includes the application of various strategies, such as acknowledging ruptures, explaining rationales, clarifying obstacles, discussing alternatives, redirecting attention, and making modifications (Muran, 2022). Other similar mixed-method efforts (quantitative and qualitative) have sought to elucidate the processes by which alliance ruptures are repaired (Hill, 2010; Muran, 2019) and to train therapists to better recognize and resolve ruptures (e.g., Muran et al., 2018; see Eubanks et al., 2019, for a meta-analysis).

The present article aims to provide further understanding of how repairing alliance ruptures can be tailored to the individual patient to improve treatment outcome across theoretical orientations. It has been suggested that any change mechanism in treatment, including the alliance, consists of distinct trait-like and state-like components (Zilcha-Mano, 2021). The trait-like component of alliance refers to individual differences between patients in their ability to form satisfying relationships with others, which may translate into an ability to form satisfying alliances with their therapists as well. The state-like component refers to within-individual changes in alliance strength occurring from one moment of the therapy session to the next (Zilcha-Mano, 2017). A synthesis of the available empirical literature disentangling the trait-like and state-like components of alliance suggests that its trait-like component is a product of the patients’ and therapists’ trait-like characteristics. By contrast, the state-like component of alliance is associated with in-treatment therapeutic processes, such as corrective relational experiences between patients and their therapists (Zilcha-Mano & Fisher, 2022).

## The Proposed Individual-Specific Framework for Repairing Alliance Ruptures

Integrating the rich theoretical, clinical, and empirical literature on repairing alliance ruptures (Muran & Eubanks, 2020a; Safran & Muran, 2000) with the trait-like state-like model (Zilcha-Mano, 2021), we proposed an individual-specific framework for repairing alliance ruptures. The proposed framework is based on our experience as clinicians, trainee supervisors, and researchers. In the proposed transtheoretical framework, at the intake sessions, the therapist evaluates the trait-like tendencies of the patient to face ruptures in interpersonal relationships. This evaluation process may result in a systematic formulation of the individuals’ main interpersonal strengths and weaknesses (Zilcha-Mano, 2024). The resulting formulation can then serve as a guide for selecting whether and how to implement a treatment or a module for repairing alliance ruptures. The evaluation includes the frequency of such ruptures, their nature and intensity, generalizability across types of relationships, as well as the patient’s awareness of the ruptures and insight regarding their origins. These trait-like tendencies can be assessed both through the collection of specific relational episodes occurring outside the therapy room, in the individual’s daily life, and based on the in-session relational moment-to-moment interactions between patient and therapist in the therapy room. An example of a structured interview that can be used to collect specific relational episodes occurring outside the therapy room is the Self-Understanding of Interpersonal Patterns Scales–Interview (SUIP-I; Gibbons & Crits-Christoph, 2017; Yaffe-Herbst et al., 2023). The SUIP-I is a semi-structured interview, in which patients are asked to share five stories about relational exchanges with significant others that they view as problematic. Structured questions are used to give patients the opportunity to verbalize their understanding of each interaction without leading them. The interviewer evaluates the patients’ ability to recognize, understand, and describe their conflictual pattern. The information collected using the SUIP-I can be used to formulate the trait-like tendencies to show ruptures, their nature, severity, and manifestations, their generalizability across types of relationships and the patient's awareness of the ruptures and insight regarding their origins. Other measures that can be implemented to explore the patients’ trait-like tendencies may include structured interviews of personality disorders (e.g., Structured Interview for DSM-IV Personality; Pfohl et al., 1997) and self-report questionnaires of interpersonal problems (e.g., Horowitz et al., 1988).

Following this initial relational-based evaluation, the therapist can develop a therapeutic program tailored to the individual patient’s relational needs, difficulties, and capabilities. The key decision about the therapeutic program in this context is whether a rupture and repair manual should be implemented as the recommended treatment (Type A below), a module augmenting another treatment manual should be used (Type B), or no rupture resolution work is needed (Type C).

During the pre-treatment evaluation phase, the therapist seeks to map the trait-like capacities of the patient to negotiate interpersonal needs. Overall, three main “types” of such trait-like abilities can be delineated. *Type C – Mature*: Individuals with mature capabilities to negotiate interpersonal needs are able to form satisfying intimate relationships with others, which balance needs for agency and communion. Across their interpersonal interactions, those individuals form satisfying relationships in which both their needs for agency and for communion are met most of the time. These individuals come to treatment with relational strengths and may not require deep relational therapeutic work. Their therapists can build on those relational strengths and invest in strengthening other mechanisms of changes, such as adaptive cognitive schemas. They can build on the patient’s abilities to form a strong helpful alliance early in the course of treatment, and maintain it throughout with little or no direct work on building relational capabilities during treatment. Few ruptures are expected with these patients during the course of treatment, and they are likely to be easily repaired using surface-based repairing techniques (e.g., explaining misunderstanding).

*Type B – Functioning but with some struggles*: Some patients are able to form satisfying relationships with others but are still struggling to meet their needs for agency or communion. They may either be able to establish and maintain relatedness with others but struggle with self-definition and individualization, or may be able to maintain autonomy but at the cost of relatedness with others. These individuals may form a good enough alliance with their therapist early in treatment with some investment by the therapist in the form of implementing active supportive techniques of alliance formation. Later in the course of treatment, when challenges appear, because of the patient’s frustrations in the process of building a helpful intimate relationship with the therapist, because of other struggles resulting from the therapeutic process, or because of interpersonal struggles outside of the therapy room, a rupture and repair module may be required to augment the treatment. The concrete points in which challenges occur may differ as a function of the type of treatment, treatment duration, patient and therapist characteristics, the fit between them, etc. For example, in short-term supportive-expressive psychodynamic treatment, some patients may face challenges at the end of the treatment, when by definition, the therapists can no longer actualize the patients’ interpersonal needs and therefore may be seen as a rejecting other (Ben David-Sela et al., 2020). Such an augmentation module is aimed at facilitating state-like improvements in the individual’s ability to negotiate interpersonal needs (e.g., Castonguay et al., 2004). Each episode in which the therapist implements techniques for repairing alliance ruptures results in state-like strengthening of the patients’ ability to negotiate their interpersonal needs. Similarly to Fredrickson’s (2004) broaden-and-build model of positive emotions, the repetitive state-like improvements in the individual’s ability to negotiate their interpersonal needs in the alliance with the therapist result in a gradual improvement in the individual’s trait-like ability to negotiate interpersonal needs. Over time, this expanded interpersonal negotiation repertoire builds useful skills and psychological resources that become available for the individual in any interpersonal interaction.

*Type A – Major interpersonal struggles*: Some patients either come to treatment because of severe interpersonal struggles or the interpersonal struggles are main contributors to their suffering and the mechanisms underlying their anxiety, depression, or other mental health symptoms. Therapists may initially perceive the formation of a strong alliance that can support the work of treatment as an unattainable goal. These patients may distrust the therapist’s goodwill or ability to help. Any therapeutic intervention the therapist may wish to implement, such as challenging thoughts or implementing a behavioral hierarchy of goals, may become an interpersonal struggle with the patient. Assigning such patients to treatment containing a mixture of both indirect and direct techniques for repairing alliance ruptures (Safran & Muran, 2000) may be more effective. The implementation of indirect techniques may produce a corrective relational experience in which, during the rupture, the therapist identifies the patient’s suffering and distress and uses implicit techniques (e.g., changing therapy tasks) that help the patient feel understood, appreciated, and validated. Through the implementation of direct techniques for negotiating interpersonal needs in the therapist-patient relationship, the patients learn, by direct participation, how interpersonal needs can be expressed and negotiated.

For individuals belonging to Types B and A, the recommended techniques should be based on the therapists’ evaluation of whether the trait-like interpersonal difficulties are of the self-definition or relatedness kind. Based on Safran and Muran (2000, 2006), it is suggested that individuals may differ in their trait-like tendencies to cope with the tension between needs for agency and for relatedness. Different trait-like interpersonal tendencies are expected to be reflected in the tendencies to show withdrawal vs. confrontational ruptures. Safran and Muran (2000) provided elaborated models of how to work with each that can help choose the right approach and technique for repairing ruptures, according to their type. For example, when the needs for agency are generally not being met in the patients' lives, and the patients may tend to respond to the therapists with withdrawal ruptures, a stage of qualified assertion may follow a stage of disembedding and attending to the rupture, which may finally result in an increase in self-assertion.

## Summary and Future Research

The proposed individual-specific framework for repairing alliance ruptures is based on theoretical conceptualizations and empirical findings. But it has never been tested directly, therefore clinical trials are needed in which patients are assigned to treatment according to their receptive “type.” Although the proposed typology refers to relatively stable trait-like characteristics of individuals, it may also be subject to change as a result of transformative and formative experiences. It is important to take into account that therapists have their own trait-like tendencies toward self-definition vs. relatedness, which can be used to better match patients with therapists (e.g., Constantino et al., 2021). Alternatively, this information can be used to tailor therapists’ training to focus on their trait-like interpersonal tendencies and the ways in which those tendencies are reflected in their interactions with their patients and affect them, as may be manifested, for example, in therapist-initiated ruptures or unsuccessful repair efforts.

After being empirically validated, the proposed typology can be further elaborated to account for and be sensitive to the socio-cultural experiences and characteristics of individuals. Its utility should also be evaluated for children, after making the relevant adaptations. For the proposed conceptualization to become useful for clinical practice, after its merit and utility receive empirical support, it should be incorporated into the skill set imparted to trainees in training programs. Given the great consistency in empirical findings showing the importance of a strong alliance for treatment success, many training programs worldwide are already teaching their trainees how to identify ruptures and repair them. Therapists trained in such programs already possess these skills, and the added value of the typology lies in how to integrate these skills into their practice and how to tailor the implementation of the skills to the individual patient’s characteristics using the proposed typology. It is recommended that all programs teach these skills, given the cumulative empirical evidence concerning the adverse effects of unrepaired ruptures and the therapeutic effects of those that are repaired. Helpful resources are available to support a large-scale implementation (e.g., Eubanks-Carter et al., 2015; Muran & Eubanks, 2020a; Muran et al., 2010).
